# Acute Abdominal Pain and Rapidly Accumulating Ascites as an Unusual Presentation of Salmonella Typhi: A Case Report

**DOI:** 10.7759/cureus.60217

**Published:** 2024-05-13

**Authors:** Joumana Samaha, Hassan E Abdulla, Renad AlSubaie, Sara Albunyan, Lina A AlMudayris

**Affiliations:** 1 Department of Internal Medicine, Al Ahsa Hospital, Al Ahsa, SAU; 2 Department of Medicine and Surgery, King Faisal University, Al Ahsa, SAU; 3 College of Medicine, King Faisal University, Al Ahsa, SAU

**Keywords:** unusual presentation, ascites, abdominal pain, salmonella, s. typhi

## Abstract

*Salmonella typhi* (*S. typhi*) infections typically present with fever and gastrointestinal symptoms. This case report on *S. typhi* enteritis documents atypical clinical, radiological, and endoscopic findings raising diagnostic challenges.

A 31-year-old male in the Kingdom of Saudi Arabia (KSA) presented with severe abdominal pain, vomiting, bloody diarrhea, and no fever. Initial diagnosis included amebiasis and other gastroenteritis infections. Despite treatment with ciprofloxacin and metronidazole, the patient's condition did not improve, and he kept having intractable abdominal pain and vomiting. Subsequent investigations, including abdominal ultrasound and esophagogastroduodenoscopy, revealed extensive and rapidly progressive intestinal inflammation with wall thickening and ascites. Stool culture eventually identified a multidrug-resistant strain of *S. typhi*, sensitive only to ceftriaxone. Treatment with ceftriaxone and continuous infusion of proton pump inhibitor (PPI) led to significant improvement.

The absence of fever in the context of bloody diarrhea, and the rapid development of ascites not improving with first-line treatment of gastroenteritis, led to the search for other diagnoses such as inflammatory bowel syndromes or tuberculosis. The presentation of diffuse intestinal wall thickening with intractable vomiting, bloody diarrhea, and progressively increasing ascites is not frequently encountered with *S. typhi*. The case also underscores the growing concern of antibiotic-resistant *S. typhi* strains. The patient's response to targeted antibiotic therapy emphasizes the importance of accurate microbial identification and susceptibility testing in managing infectious diseases.

This case report illustrates an atypical presentation of *S. typhi* enteritis with progressively increasing ascites and increased intestinal wall thickening. The uncommon complicated clinical picture led to challenges in diagnosis and management. It emphasizes the need for high clinical suspicion and comprehensive diagnostic approaches in atypical cases of common infections, especially in the context of increasing antibiotic resistance.

## Introduction

*Salmonella typhi* (*S. typhi*), a gram-negative bacterium of the Enterobacteriaceae family, has long been a threat to human health, inflicting a substantial burden worldwide [1]. This pathogen has effectively adapted to the human host employing a variety of complex virulence mechanisms to generate a long-lasting and frequently crippling infection [2,3]. In 2019, an updated modeling study estimated that 9.2 million typhoid fever cases were reported, leading to roughly 110,000 deaths [3]. According to the Saudi Ministry of Health (MOH), there were 6.81 cases of *Salmonella *infection per 100,000 of the population in 2021 [4].

Transmission of *S. typhi* occurs from human-to-human, predominantly through fecal-oral transmission, often facilitated by the consumption of water contaminated with the pathogen. Effective prevention strategies for typhoid include vaccination, ensuring access to drinking water, and enhancing sanitation practices. Additionally, antibiotics play a pivotal role in the treatment of typhoid; however, it is noteworthy that the emergence of antibiotic-resistant strains of *S. typhi *has witnessed a concerning surge in prevalence [5]. In the context of *Salmonella* infections, the emphasis has generally been on non-typhoidal *Salmonella *serotypes, which are usually linked with self-limiting gastroenteritis [6]. *Salmonella typhi*, on the other hand, is distinctive for its capacity to induce systemic sickness marked by prolonged fever, abdominal pain, and possibly life-threatening complications [1].

This case report details a recent encounter with* S. typhi *enteritis, with unusual presentation of severe abdominal pain, vomiting, bloody diarrhea, and rapidly accumulating ascites, giving rise to diagnostic challenges, in addition to the strain being highly resistant.

## Case presentation

**Figure 1:**
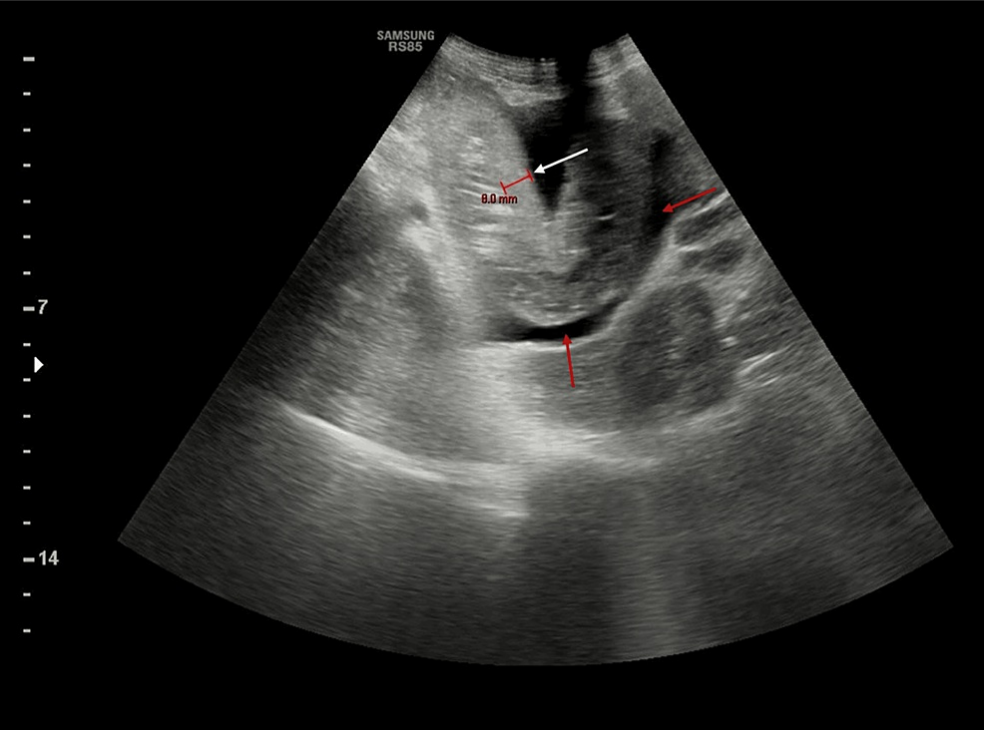
First abdominal ultrasound showing ascites (red arrow) and intestinal wall thickening (white arrow)

**Figure 2:**
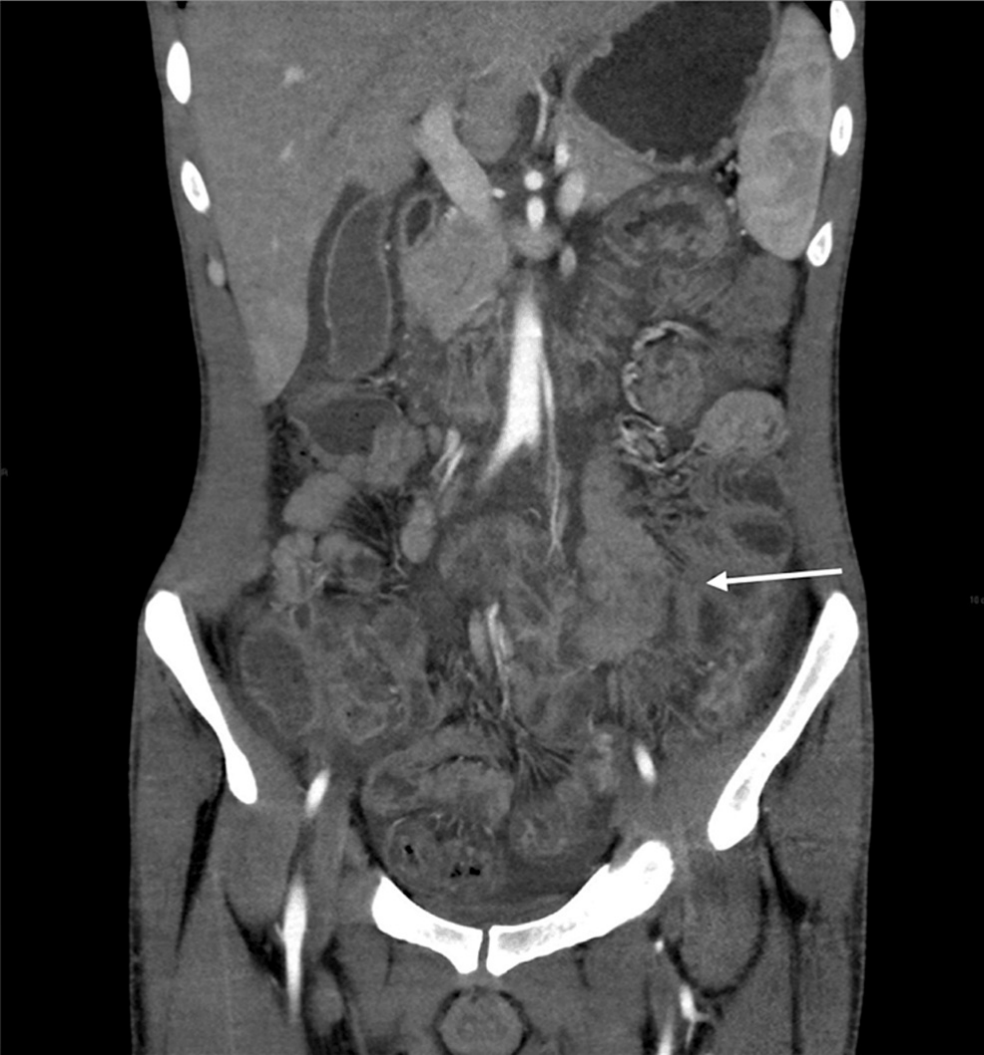
Abdominal CT showing intestinal wall thickening (white arrow)

A 31-year-old Pakistani male, residing for more than a year, in Al Hofuf city, Al Ahsa, Eastern region, KSA, presented to the emergency department with an alarming episode of acute abdominal pain and intense vomiting followed by bloody diarrhea over a few days. Notably, the clinical presentation was devoid of febrile symptoms, which often accompany such gastroenterological manifestations. His medical history was unremarkable except for this acute presentation. The patient's physical examination was significant for dehydration and severe diffuse abdominal tenderness, but he was hemodynamically stable and was assessed by a surgeon who excluded any surgical acute abdomen. Diagnostic endeavors were initiated with a stool analysis, which yielded numerous red blood cells (RBCs) and the presence of Entamoeba histolytica, trophozoites, and cysts. The other laboratory investigations showed unremarkable complete blood count (CBC), C-reactive protein (CRP), erythrocyte sedimentation rate (ESR), amylase, and lipase. Liver function test (LFT) showed elevated bilirubin at 1.8 mg/dL and decreased albumin at 2.6 g/dL, attributed to severe vomiting and low food intake. The stool for occult blood was positive. Stool culture was taken. Radiological assessments, including an abdominal ultrasound followed by a CT scan, were undertaken to gain deeper insights into the patient's internal landscape. The visuals highlighted mild ascites and prominent circumferential wall thickening, dominantly involving the jejunal and distal ileal loops, and other organs had a normal appearance, mainly the liver, and spleen (Figures 1, 2). The radiology report revealed small bowel loops infectious/inflammatory process, i.e., enteritis.

Initial diagnosis of this bloody enteritis included amebiasis, other infectious gastroenteritis, and noninfectious causes such as inflammatory bowel disease, and the least likely differential was malignancy because of the acute characteristic of the presentation.

The patient did not improve on ciprofloxacin and metronidazole. He was still having abdominal pain, vomiting, and bloody diarrhea. His abdominal pain was precipitated by minimal food intake, even fluids were not tolerate, and, thus, he stopped eating. Initial treatment strategies included hydration, analgesics, proton pump inhibitor, anti-emetic, and an initial empiric regimen of ciprofloxacin (400 mg IV every 12 hours) maintained for two days. This was supplemented with metronidazole (500 IV every 8 hours), a mainstay for protozoal infections, which was continued for the duration of his hospitalization around 10 days targeting the Entamoeba histolytica found in the stool analysis.

However, after 48 hours of initial management, the patient's clinical trajectory did not veer toward improvement, necessitating further diagnostic investigations. Given the non-responsiveness to ciprofloxacin and metronidazole, and the clinical deterioration of the patient who was still having abdominal pain, vomiting, bloody diarrhea and he stopped eating due to severe abdominal pain precipitated by minimal food intake, even fluids were not tolerated. His leucocyte count, which was normal on admission, increased to 18300/mm³, CRP increased to 7 mg/dL, albumin decreased further to 1.7 g/dL, and fecal calprotectin elevated to 738 µg/g.

**Figure 3:**
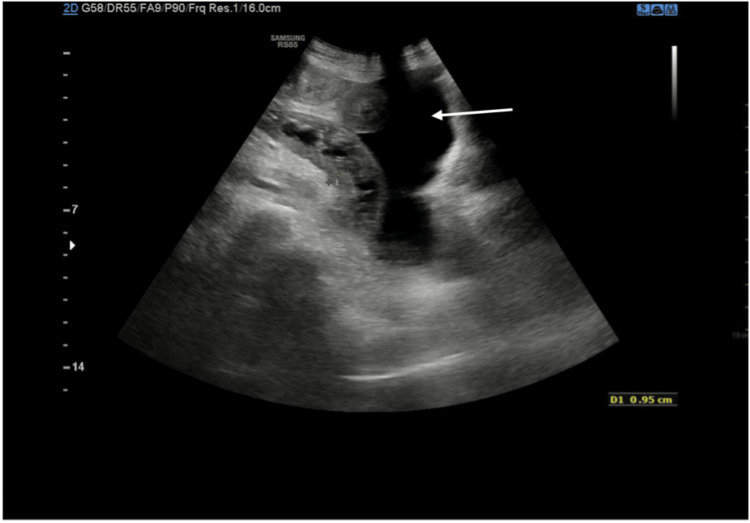
Follow-up abdominal ultrasound showing increased ascites and abdominal wall thickening

A follow-up ultrasound of the abdomen showed progression of the abdominal ascites from mild to moderate amount with increased small bowel loops circumferential wall thickening involving the jejunal and ileal loops (maximum thickening at the jejunal loops measuring 1.1 cm) (Figure 3). The gallbladder was distended, measuring 9.3 cm in length, with minimal sludge and mild diffuse circumferential wall thickening of 3 mm mostly reactive from ascites. At that time, an ultrasound-guided procedure successfully aspirated 60 mL of ascitic fluid. Ciprofloxacin was substituted with piperacillin/tazobactam (4.5 g every 8 hours), which was kept diligently for five days. The antibiotic carousel was continued with the introduction of levofloxacin (750 mg IV daily for five days).

The result of the peritoneal fluid analysis showed bloody fluid, with a negative gram stain and culture. The serum ascitic albumin gradient (SAAG) was <1.1 g/dL, which was suggestive of exudate, which ruled out portal venous occlusion (i.e., Budd Chiari syndrome) as a possible cause of ascites, and confirmed our suspicion of the infectious process as the first diagnosis, whether bacterial or mycobacterial, and then malignancy as the second possibility. Carcinoembryonic antigen (CEA), carbohydrate antigen 19-9 (CA 19-9), and angiotensin-converting enzyme (ACE) were normal. Furthermore, acid-fast bacilli (AFB) and molecular testing for tuberculosis (TB) (XpertTB-Rif test) were ordered on the peritoneal fluid.

**Figure 4:**
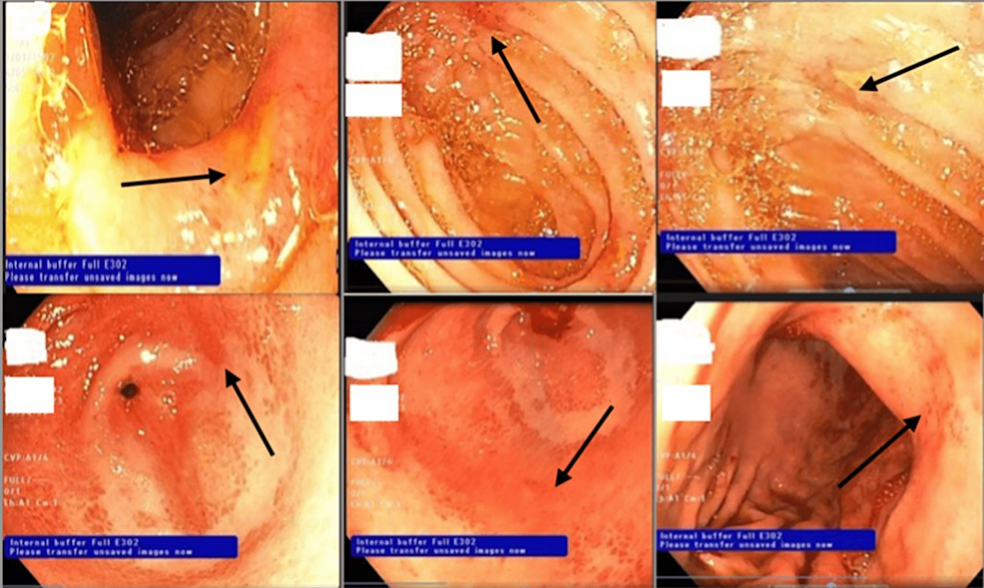
Gastroscopy showing gastrointestinal ulceration

The patient's diagnostic voyage culminated in esophagogastroduodenoscopy in light of his persistent severe abdominal pain, recurring vomiting episodes, and inability to take any food or fluid. Endoscopic visualization reported a detailed picture of his gastric milieu, showing body linear superficial erosions with marked erythema along most of the gastric fold. The antrum showed severe superficial gastropathy and marked erythema with superficial ulceration that affected most of the antral mucosa with alternating normal mucosa, which was biopsied for *Helicobacter pylori*. Duodenum visualization showed superficial erythema of the bulb, and diffuse edematous and erythematous mucosal lining of the second part. Hardly the scope was introduced into the first part of the jejunum in which large multiple deep ulcers with everted edges were seen, and samples for biopsy were taken to rule out a potential TB infection, and were sent for AFB, mycobacterial culture, and molecular testing for TB (XpertTB-Rif test) (Figure 4). This endoscopic picture of protein-losing enteropathy explained the rapidly decreasing albumin level and hence increasing ascites. Therefore, the patient was kept nil per os (NPO), with continuous infusion of omeprazole 8 mg/h for 72 hours.

Later on, the stool culture revealed *S. typhi*, resistant to ampicillin, ciprofloxacin, levofloxacin, trimethoprim/sulfamethoxazole, exclusively sensitive to ceftriaxone, which, was immediately prescribed at a dose of 2g IV every 12 hours, and induced significant clinical improvement after only 24 hours. However, his improvement was not only due to the targeted antibiotic administration but also due to the patient being kept NPO, with omeprazole infusion, which contributed to the healing of the injurious effect of *Salmonella* on the gastrointestinal mucosa. His feeding improved, as well as his laboratory results, leucocyte count, albumin, and fecal calprotectin (which decreased markedly to 113 µg/g).

The rest of the investigation results came later as follows: histopathology report of the endoscopic biopsy from the gastric body yielded moderate chronic non-specific gastritis with mild activity, the gastric mucosa sections unveiled small clusters of plasma cells with occasional intraepithelial neutrophils, no *H. pylori* was detected, and there was an absence of dysplasia, malignancy, and intestinal metaplasia. There was a notable absence of goblet cells. While the epithelium showed normal maturation, the lamina propria was marked by edema and was populated by a mix of non-specific chronic inflammatory infiltrate and neutrophils. The entire histological panorama indicated an infected and inflamed gastric environment, devoid of any malignant or dysplastic transformation.

The TB studies were all negative, including AFB, mycobacterial culture, molecular testing for TB (XpertTB-Rif test), and purified protein derivative. The QuantiFERON gold test was indeterminate. The complete clinical recovery ruled out inflammatory bowel disease and confirmed the sole etiology of *S. typhi *for this severe enteritis.

Upon discharge, the patient was prescribed oral cefixime (400 mg daily for six days to complete a total of 10 days) to continue his treatment for *S. typhi *enteritis, coupled with metronidazole 200 mg/diloxanide furoate 250 mg (two tablets every 8 hours for 10 days) to counteract the *Entamoeba* cysts, in addition to omeprazole as treatment for the gastrointestinal ulcerations.

## Discussion

*Salmonella typhi* causes typhoid fever, an enteric illness, which, like many febrile infections, causes vague symptoms [7]. Patients often develop symptoms after an incubation period of 7 to 14 days following consumption of food or water contaminated with* S. typhi* [8]. Usually, the symptoms include fever, chills, bradycardia, abdominal pain, nausea, vomiting, watery diarrhea or constipation, lack of appetite, and weight loss [9]. However, our case describes a rare, atypical presentation of *S. typhi* infection that caused a dilemma in the diagnosis. Contrary to the primary feature of this infection, high-grade fever [10], our patient presented with normal body temperature. In addition, the patient’s presentation was especially intriguing due to the presence of ascites rapidly accumulating, along with the severe enteritis manifested by the intestinal wall thickening. These findings raised the suspicion of TB, which made the diagnosis more challenging, especially because of the high incidence of TB in KSA and the nationality of the patient being Pakistan. Middle East is one of the endemic areas of TB, with incidence rates of 14 and 18 per 100,000 people in the KSA and Bahrain, respectively [11]. The Hajj and Umrah pilgrimages, as well as the huge percentage of expatriates coming from Asia and Africa where the prevalence of TB is high, are the main reasons for this ongoing TB burden in KSA [12].

Normally, a few days after receiving empirical antibiotic therapy, the majority of patients with typhoid fever improve. In stark deviation from this conventional narrative, our patient's clinical progression post-antibiotic initiation did not align with the anticipated recuperation, adding more intricacies and dilemmas to the atypical clinical manifestations. These organisms have demonstrated resistance to first-line medications, including trimethoprim-sulfamethoxazole, ampicillin, and chloramphenicol. As a result, the main options for treatment are fluoroquinolones, third-generation cephalosporins, and azithromycin. In addition, *S. typhi *has become resistant also to a variety of antibiotics throughout time, giving rise to MDR (multi-drug resistant) and XDR (extended-drug resistant) *S. typhi *[13]. In this case, the patient was resistant to ampicillin, ciprofloxacin, levofloxacin, trimethoprim/sulfamethoxazole, and sensitive to ceftriaxone.

Moreover, complications from *S. typhi* do arise in some patients, affecting any organ system. Hepatosplenomegaly, jaundice, gastrointestinal bleeding, intestinal perforation, and acalculous cholecystitis are some of the serious complications of typhoid fever [14]. In our patient, *S. typhi* resulted in severe gastrointestinal ulcerations and bleeding, which are best treated by continuous IV infusion of omeprazole [15]. Additionally, our patient had increasing ascites due to this protein-losing enteropathy and hypoalbuminemia.

*Salmonella typhi*, beyond causing acute conditions, can cause a chronic carrier state and has the propensity to trigger prolonged infections of the gallbladder, lasting over 12 months, in an estimated 3-5% of individuals following acute clinical or subclinical episodes [16]. Antibiotics alone have conventionally served as a treatment approach for typhoid cholecystitis. However, it is pivotal to note that certain scenarios, such as the presence of gallbladder stones, which increases the risk of chronic carriage, or the manifestation of fulminant cholecystitis accompanied by sepsis, render conservative antibiotic therapy alone unsuitable [17]. In such instances, cholecystectomy becomes imperative. Reflecting on our patient's case, a follow-up was scheduled to redo the abdominal ultrasound, re-evaluate the gallbladder sludge, and advise cholecystectomy.

## Conclusions

In conclusion, this case report highlights the challenging diagnosis and the atypical presentation of *S. typhi* infection, which initially mimicked TB, emphasizing the complexity in identifying such cases, especially in regions with a high burden of TB such as the Middle East. The absence of fever and the presence of progressive ascites and severe enteritis, in addition to the absence of improvement of the patient to the first-line antibiotics given, posed challenges in diagnosing and managing this case. The rise of *S. typhi* strains that are MDR illustrates the significance of receiving adequate antibiotic treatment. The risk of typhoid cholecystitis and the requirement for surgical intervention, such as cholecystectomy, further highlight the individualized treatment plans for typhoid fever complications. This report serves as a reminder of the diverse clinical manifestations and management challenges posed by this infectious disease.
